# Impact of Sarcopenia as a Prognostic Biomarker of Bladder Cancer

**DOI:** 10.3390/ijms19102999

**Published:** 2018-10-01

**Authors:** Hiroshi Fukushima, Kosuke Takemura, Hiroaki Suzuki, Fumitaka Koga

**Affiliations:** Department of Urology, Tokyo Metropolitan Cancer and Infectious Diseases Center Komagome Hospital, 3-18-22 Honkomagome, Bunkyo-ku, Tokyo 113-8677, Japan; fukuuro@tmd.ac.jp (H.F.); takemura-urol@cick.jp (K.T.); smu.udm@gmail.com (H.S.)

**Keywords:** sarcopenia, prognosis, biomarker, bladder cancer, urothelial carcinoma

## Abstract

Sarcopenia, the degenerative and systemic loss of skeletal muscle mass, indicates patient frailty and impaired physical function. Sarcopenia can be caused by multiple factors, including advanced age, lack of exercise, poor nutritional status, inflammatory diseases, endocrine diseases, and malignancies. In patients with cancer cachexia, anorexia, poor nutrition and systemic inflammation make the metabolic state more catabolic, resulting in sarcopenia. Thus, sarcopenia is considered as one of manifestations of cancer cachexia. Recently, growing evidence has indicated the importance of sarcopenia in the management of patients with various cancers. Sarcopenia is associated with not only higher rates of treatment-related complications but also worse prognosis in cancer-bearing patients. In this article, we summarized metabolic backgrounds of cancer cachexia and sarcopenia and definitions of sarcopenia based on computed tomography (CT) images. We conducted a systematic literature review regarding the significance of sarcopenia as a prognostic biomarker of bladder cancer. We also reviewed recent studies focusing on the prognostic role of changes in skeletal muscle mass during the course of treatment in bladder cancer patients. Lastly, we discussed the impact of nutritional support, medication, and exercise on sarcopenia in cancer-bearing patients.

## 1. Introduction

Bladder cancer is the most common malignancy of the urinary tract in the world, with approximately 430,000 new cases and 165,000 deaths each year [1]. The major histology of bladder cancer is urothelial carcinoma. Based on the pathological depth of tumor invasion, bladder cancer is classified into two groups: non-muscle-invasive bladder cancer (NMIBC) and muscle-invasive bladder cancer (MIBC). NMIBC is treated with bladder-preserving treatments, including transurethral resection of the bladder tumor and intravesical instillation therapy [2]. Patients with MIBC generally require total cystectomy and urinary diversion as a curative treatment [3]. However, approximately half of MIBC patients undergoing total cystectomy die within five years because MIBC is potentially an aggressive disease and frequently progresses to a metastatic disease postoperatively [4]. Once MIBC patients develop distant metastasis, their prognoses are poor despite receiving systemic chemotherapy with a median overall survival (OS) of approximately 15 months [5]. Thus, bladder cancer is still a challenging disease, although the recent advent of immuno-oncology drugs is shifting the paradigm of the management of bladder cancer patients [6]. Pre-therapeutic risk assessment based on prognostic biomarkers can help clinicians to predict their outcomes and counsel patients about treatment options. Therefore, identifying prognostic biomarkers contributes to better management for bladder cancer patients.

Sarcopenia is a syndrome representing the degenerative and systemic loss of skeletal muscle mass [7]. According to recent surveys, the prevalence of sarcopenia is relatively high, ranging from 15% at 65 years to 50% at 80 years [8]. Variations in genes, such as *MSTN*, *VDR*, and *ACE*, determine the variability in skeletal muscle phenotype and the prevalence of sarcopenia in an elderly population [9]. Sarcopenia is associated with lower physical activity, morbidity, and mortality [10,11]. Sarcopenic patients tend to have higher morbidity from infectious diseases [12], metabolic syndrome [13], insulin resistance [14], and cardiovascular diseases [15]. Sarcopenia is pathophysiologically associated with various etiologies, including advanced age, lack of exercise, poor nutritional status, inflammatory diseases, and endocrine diseases [7]. Malignant diseases can also cause sarcopenia [16]. In patients with cancer cachexia, anorexia, poor nutrition, and systemic inflammation make the metabolic state more catabolic, resulting in sarcopenia [17]. Therefore, sarcopenia is considered as one of manifestations of cancer cachexia.

Recent studies have shown the prognostic impact of sarcopenia in various cancers. Sarcopenic patients show significantly worse survival than non-sarcopenic counterparts with lung or gastrointestinal cancer [18,19], hepatic cell carcinoma [20], esophageal cancer [21], lymphoma [22], melanoma [23], or renal cell carcinoma [24,25]. In bladder cancer, the role of sarcopenia in predicting survival has been clarified. In this article, we summarized metabolic backgrounds of cancer cachexia and sarcopenia and definitions of sarcopenia based on computed tomography (CT) images. Moreover, we conducted a systematic literature review on published studies to summarize comprehensively the current clinical evidence on the prognostic role of sarcopenia in bladder cancer patients. We also reviewed recent studies focusing on the prognostic importance of changes in skeletal muscle mass during the course of treatment in bladder cancer patients. Finally, we discussed the impact of nutritional support, medications, and exercise on cancer cachexia and sarcopenia in cancer-bearing patients.

## 2. Metabolic Background of Cancer Cachexia and Sarcopenia

Cancer cachexia is a multifactorial syndrome characterized by progressive weight loss, which is due to the depletion of adipose tissue and skeletal muscle mass. In the early phase of cancer cachexia, adipose tissue is depleted [26]. Skeletal muscle wasting is promoted after the progression of cancer cachexia [27]. Anorexia, which is caused by cancer itself or treatment for cancer, is frequently observed in patients with cancer cachexia. Moreover, resting energy expenditure increases in patients with cancer cachexia, leading to the progressive loss of body weight [28]. In the process of the progression of cancer cachexia, lipolysis and fatty acid oxidation are activated in skeletal muscle, whereas glycolysis is suppressed [29,30]. Increased oxidative stress caused by up-regulated fatty acid oxidation can contribute to skeletal muscle wasting [30]. Several mechanisms, including epinephrine stimulation and increased secretion of cytokines, are involved in these metabolic changes [31]. Moreover, skeletal muscle depletion is caused by increased protein degradation mainly by the activated ubiquitin-proteasome pathway, in which multiple receptor-mediated signaling pathways are involved [27]. In this section, we summarize the metabolic changes and the mechanisms of the depletion of adipose tissue and skeletal muscle mass during cancer cachexia.

### 2.1. Adipose Tissue Depletion in Cancer Cachexia

Adipose tissue volume decreases in the early process of cancer cachexia [26]. The breakdown of adipose tissue is caused by lipolysis of triglyceride, which is mediated by adipose triglyceride lipase (ATGL) and hormonal-sensitive lipase (HSL) [32]. In a previous study of ATGL- or HSL-deficient animal models, the absence of ATGL and, to lesser degree, HSL reduces fatty acid mobilization and adipose tissue loss, leading to maintained skeletal muscle mass, suggesting that excessive depletion of adipose tissue may be involved in the progression of skeletal muscle atrophy [29]. Up-regulation of lipolysis is induced by various factors, including enhanced stimulation of β-adrenergic receptor, increased secretion of cytokines such as tumor necrosis factor (TNF)-α, interleukin (IL)-1, IL-6, and IL-8, and increased expression of lipid-mobilizing factors, such as zinc-α2 glycoprotein-1 (AZGP1) [32]. White adipose tissue browning, which is associated with increased expression of uncoupling protein 1 (UCP1), increases thermogenesis and energy expenditure during cancer cachexia [28]. This process is also affected by β-adrenergic receptor stimulation and cytokines such as TNF-α and IL-6 [28].

### 2.2. Skeletal Muscle Depletion in Cancer Cachexia

Skeletal muscle depletion occurs as a consequence of reduced protein synthesis and increased degradation of proteins in the late phase of cancer cachexia [27]. Reduced protein synthesis can be caused by low nutritional status as a result of anorexia and decreased food intake [27,32]. Skeletal muscle protein degradation is promoted mainly by the ubiquitin-proteasome pathway, which is induced by myostatin, activin A, cytokines such as TNF-α and IL-6, and proteolysis-inducing factor [31,32]. Myostatin and activin A, members of the transforming-growth factor β (TGF-β) family, bind activin type 2 receptor B (ActR2B) and activate Smad2/3 and p38 mitogen-activated protein kinase (MAPK) signaling, resulting in the up-regulation of Atrogin-1 and the muscle ring finger protein 1 (MuRF-1), which are muscle-specific E3 ligases and play roles as key regulators of ubiquitin-driven protein degradation in the skeletal muscle [27,31]. Moreover, the phosphatidylinositol-3 kinase (PI3K)/Akt/mammalian target of rapamycin (mTOR) pathway and forkhead box O (FOXO), which are general regulators of skeletal muscle mass homeostasis, are affected in cancer cachexia [31,32,33]. TNF-α up-regulates Atrogin-1 by increasing nuclear FOXO4 protein in skeletal muscle [34]. Glucocorticoid receptor regulates the expression of Atrogin-1 and MuRF-1 [27,31]. The binding of insulin-like growth factor-1 (IGF-1) to its receptor causes the activation of PI3K/Akt/mTOR pathway, which down-regulates FOXO3 and results in decreased expression of Atrogin-1 and MuRF-1 [27,31]. Taken together, various signaling pathways are related to the regulation of skeletal muscle protein degradation. Their inhibition may contribute to the prevention of cancer cachexia and sarcopenia.

Oxidative stress promotes skeletal muscle wasting. Fukawa et al. revealed up-regulation of fatty acid oxidation and down-regulation of glycolysis in the skeletal muscle using transcriptomics of human muscle stem cell-based models and human cancer-induced cachexia models in mice [30]. Interestingly, they also showed that increased oxidative stress caused by excessive fatty acid oxidation could impair muscle growth [30]. Therefore, increased oxidative stress can cause sarcopenia through excessive fatty acid oxidation in the process of the progression of cancer cachexia. Inhibiting the process of fatty acid oxidation could be efficacious in preventing cancer cachexia and sarcopenia. In contrast, skeletal muscle mitochondrial oxidative capacities decrease without alteration of adenosine triphosphate (ATP) production efficiency in a rat model of cancer cachexia [35], which appears to be inconsistent with the results reported by Fukawa et al. [30], but this may contribute to lipid droplet accumulation in skeletal muscle mass [36].

## 3. Evaluation of Sarcopenia Using Computed Tomography (CT) Images

**Figure 1:**
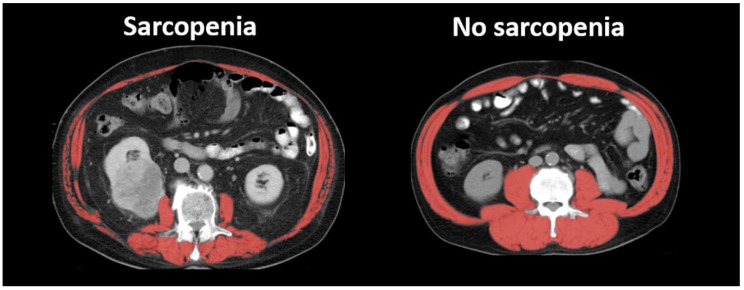
Computed tomography (CT) images of typical sarcopenic and non-sarcopenic cases. Skeletal muscle area is shown in red.

According to the European Working Group of Sarcopenia in Older People (EWGSOP), sarcopenia is determined based on three factors: lower skeletal muscle mass, lower skeletal muscle strength, and lower physical performance [7]. Skeletal muscle strength can be evaluated by upper-limb hand-grip dynamometry and lower-limb extension strength testing. The assessment of physical function is generally based on walking speed. As for skeletal muscle mass, bioimpedance analysis, anthropometry, dual energy X-ray imaging, CT, and magnetic resonance imaging (MRI) are recommended as methods to measure skeletal muscle mass by EWGSOP [7]. In cancer-bearing patients, including bladder cancer patients, CT images are generally used in the evaluation of sarcopenia, since abdominal CT scans are routinely performed for diagnosis, staging, surveillance of recurrence after treatment, and assessment of therapeutic response [37]. Therefore, most of the previous studies on sarcopenia and bladder cancer used CT images to measure skeletal muscle mass and define sarcopenia (Figure 1). In our systematic literature review below, all the articles used CT images.

### 3.1. Measurement of Skeletal Muscle Mass Using CT Images

Axial CT images at the lumbar vertebral level are used to measure skeletal muscle areas because the total lumbar-skeletal muscle cross-sectional area is linearly correlated to the whole-body skeletal muscle mass [38]. The total skeletal muscle area at the third lumbar vertebra, including the psoas, paraspinal muscles (the erector spinae and quadratus lumborum), and abdominal wall muscles (the transversus abdominus, external and internal obliques, and rectus abdominus), is measured using software such as Slice-O-Matic (Tomovision, Montreal, QC, Canada) and OsiriX imaging software (Pixmeo, Geneva, Switzerland). The cross-sectional areas of skeletal muscle are identified using Hounsfield Unit thresholds of −29 to +150.

### 3.2. Skeletal Muscle Index (SMI)

Skeletal muscle index (SMI) is used widely in evaluating sarcopenia in cancer-bearing patients. SMI is calculated by normalizing skeletal muscle area for height in meters squared, as is body mass index (BMI). Two major established definitions of sarcopenia have been proposed so far. First, the International Consensus of Cancer Cachexia (ICCC) proposed cutoff values of SMI as 55 cm^2^/m^2^ for males and 39 cm^2^/m^2^ for females [16]. Second, Martin et al., defined BMI-incorporated cutoff values of SMI as <43 cm^2^/m^2^ for males with BMI < 25 kg/m^2^, <53 cm^2^/m^2^ for males with BMI ≥ 25 kg/m^2^, and <41 cm^2^/m^2^ for females [18]. Both of the two definitions were the best cutoffs to predict overall mortality using a cohort of patients with lung or gastrointestinal cancer, and either of them has been used to define sarcopenia in most previous studies on bladder cancer [37].

### 3.3. Psoas Muscle Index (PMI)

In some previous studies, only the psoas muscle area was measured on axial CT images at the lumbar vertebral level. The psoas muscle index (PMI) is calculated by normalizing the psoas muscle area for height in meters squared. Although a correlation between PMI and whole-body skeletal muscle mass has not yet been evaluated, the strong correlation between PMI and SMI suggests that PMI also represents whole-body skeletal muscle mass [39]. Hamaguchi et al. proposed the cutoff values of PMI to define sarcopenia as 6.36 cm^2^/m^2^ for males and 3.92 cm^2^/m^2^ for females, using a cohort of adult donors for living donor liver transplantation [39]. However, because their cohort included only Japanese patients, the use of their values may be limited to Asian populations.

### 3.4. Skeletal Muscle Density

In addition to the volume of the skeletal muscle, the quality of the skeletal muscle can be evaluated on CT scan. Skeletal muscle density is determined based on the CT density (Hounsfield unit) of the skeletal muscle [40]. Lower skeletal muscle density reflects more fat infiltration in skeletal muscle mass, which is related to lower function of the skeletal muscle and lower physical performance. Moreover, increased fat infiltration in skeletal muscle mass is involved in insulin resistance [41], which decreases glucose uptake in skeletal muscles and can eventually contribute to skeletal muscle atrophy. Because increased fat infiltration in the skeletal muscle is one of the characteristics of cancer cachexia, lower skeletal muscle density is considered as an indicator of the progression of cancer cachexia [40].

## 4. Hybrid Nature of Sarcopenia as a Prognostic Biomarker

**Figure 2:**
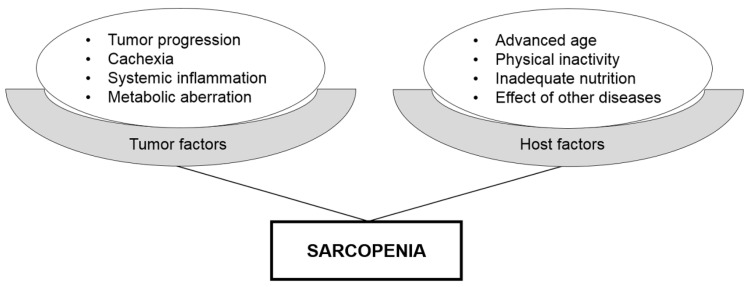
Hybrid nature of sarcopenia.

Prognostic tumor biomarkers generally reflect tumor aggressiveness, including tumor stage, histological grade, lymphovascular invasion, and patient survival. Several prognostic biomarkers are related to the general condition of the host; e.g., age, sex, performance status, BMI, anemia, etc. Notably, sarcopenia reflects both tumor and host factors (Figure 2). Because sarcopenia develops as a consequence of tumor progression, tumor-induced systemic inflammation, or metabolic aberration, its presence indicates tumor aggressiveness. In addition, sarcopenic patients are characterized by poor general health and physical performance, which can contribute to worse prognosis of cancer-bearing patients. High prognostic performance of sarcopenia could be explained by its hybrid nature, which is a unique feature as a prognostic biomarker.

## 5. Systematic Literature Review

**Figure 3:**
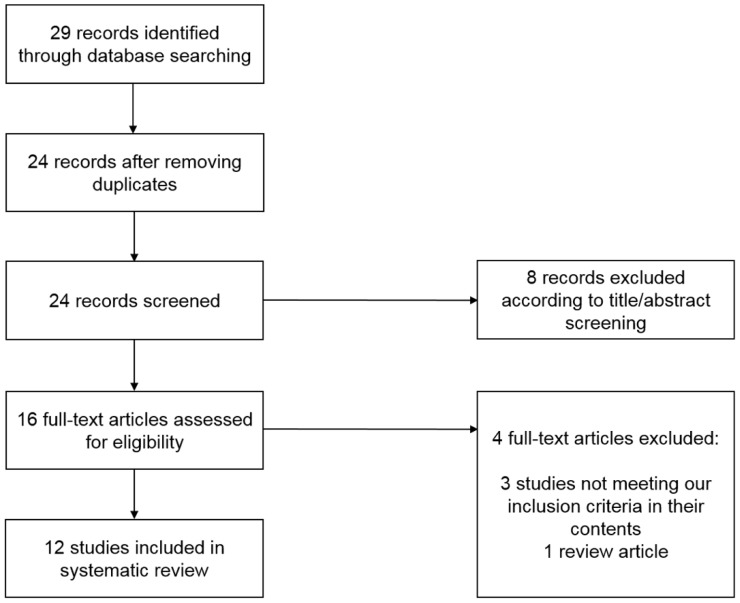
Flow diagram of systematic literature search.

A systematic literature review was performed to search for studies investigating the prognostic role of sarcopenia in bladder cancer patients according to the PRISMA guidelines [42]. The search was restricted to articles written in English and performed using PubMed, Medline, and Cochrane Libraries by entering the terms “sarcopenia and urothelial carcinoma” and “sarcopenia and bladder cancer”. Twenty-nine articles published from June 2014 to April 2018 were identified on 1 April 2018. There was no literature before June 2014 according to our literature search. Two independent investigators (H.F. and K.T.) conducted the literature search and selection of articles. Potential discrepancies were resolved by open discussion. Details of the search and article selection are summarized in the flow diagram (Figure 3). Studies were included if they were published as original articles investigating the prognostic role of sarcopenia in bladder cancer patients. Review articles, case reports, editorial comments, letters, meeting abstracts, and studies not meeting our inclusion criteria in their content were excluded. Our systematic literature review has several limitations. First, 12 articles were included in our systematic review, all of which were retrospective, and no study of level 1 evidence was included, indicating possible high risks of bias. Second, several different methods and definitions were used to evaluate sarcopenia using CT images in included studies.

## 6. Prognostic Role of Sarcopenia in Bladder Cancer

Table 1 lists published studies on the prognostic role of sarcopenia in bladder cancer patients. Most studies reported that sarcopenia was associated with worse prognosis. A systematic literature review identified six studies involving patients undergoing a radical cystectomy (due to high-risk NMIBC or MIBC) and four studies involving patients with inoperable locoregionally advanced and/or metastatic diseases. No studies investigated the association between sarcopenia and survival in low- or intermediate-risk NMIBC patients. Nine of the 10 studies used either SMI or PMI to define sarcopenia.

### 6.1. Survival after a Radical Cystectomy

Although a radical cystectomy with pelvic lymph node dissection is the standard of care for high-risk NMIBC and MIBC patients, its main problems include high incidences of perioperative complications [3]. In the contemporary radical cystectomy series, the incidence of major complications of Clavien–Dindo classification grade 3 or greater ranges from 5 to 26%, with a mortality rate of 0–3.9% [43]. Several studies showed that sarcopenia is significantly associated with higher rates of perioperative complications of a radical cystectomy [44,45].

As shown in Table 1, six studies reported the prognostic role of sarcopenia in bladder cancer patients undergoing a radical cystectomy [45,46,47,48,49,50]. Five of them revealed that sarcopenia is a significant predictor of cancer-specific survival (CSS) and OS [46,47,48,49,50]. Psutka et al., for the first time demonstrated that sarcopenia is an independent predictor for both poorer CSS and OS [46]. The 5-year CSS and OS rates were lower for sarcopenic patients than for non-sarcopenic counterparts (49% vs. 72% for CSS and 39% vs. 70% for OS, respectively). Three studies from Japan reported similar results to those of Psutka et al. [47,48,49]. Recently, a multi-center retrospective study from Germany demonstrated that sarcopenia is an independent predictor for both poorer CSS and OS in 500 bladder cancer patients undergoing a radical cystectomy [50]. Only one study, reported by Smith et al., showed no association between sarcopenia and OS [45]. This discrepant result may be due to the different methods for estimating skeletal muscle volume. Four studies calculated SMI, and three of them used the definition of sarcopenia proposed by Martin et al. However, Smith et al. calculated cross-sectional psoas muscle area using 3-dimensional computerized image analysis and defined sarcopenia using their own criteria.

Taken together, most previous studies demonstrated that sarcopenia is a significant poor prognostic factor in bladder cancer patients undergoing a radical cystectomy.

### 6.2. Survival in Inoperable Advanced Disease

Four studies evaluated the prognostic role of sarcopenia in patients with inoperable advanced bladder cancer (Table 1) [51,52,53,54]. Because upper tract urothelial carcinoma is histologically and biologically similar to bladder cancer, three of them included advanced upper tract urothelial carcinoma in their cohorts [51,52,54]. Fukushima et al. showed for the first time that sarcopenia is an independent predictor for shorter OS in patients with advanced urothelial carcinoma (inoperable locoregionally advanced disease and/or metastatic diseases to lymph nodes or distant organs) [51]. The median OS of sarcopenic patients was significantly shorter than that of non-sarcopenic counterparts (11 vs. 31 months). Taguchi et al. reported that sarcopenia is an independent predictor for shorter CSS in metastatic urothelial carcinoma patients receiving systemic chemotherapy as the first-line therapy [52]. Kasahara et al. showed the prognostic significance of sarcopenia in advanced bladder cancer patients receiving gemcitabine and nedaplatin therapy [53]. In addition, Abe et al. could not confirm the significance of sarcopenia in predicting OS, but they showed that SMI stratified by BMI was an independent predictor for shorter OS [54].

Thus, previous studies indicated that sarcopenia is a significant poor prognostic factor in inoperable advanced bladder cancer patients.

## 7. Prognostic Role of Changes in Skeletal Muscle Mass in Bladder Cancer

Because disease status and patient conditions can affect skeletal muscle mass in cancer-bearing patients, changes in skeletal muscle mass during and after treatment may represent post-therapeutic prognosis. As shown in Table 2, three studies investigated the prognostic role of changes in skeletal muscle mass during treatment in bladder cancer patients [48,55,56]. Miyake et al. reported that a 10% loss in psoas muscle volume before and after a radical cystectomy was an independent predictor for shorter OS [48]. Fukushima et al. reported that post-therapeutic skeletal muscle mass recovery was an independent predictor for both better recurrence-free survival and OS in advanced urothelial carcinoma treated with platinum-based chemotherapy as the first-line therapy [56]. Meanwhile, Zargar et al. showed that decline in psoas muscle volume during neoadjuvant chemotherapy was not predictive of OS in bladder cancer patients treated with neoadjuvant chemotherapy and a radical cystectomy [55].

Although limited data suggest the prognostic significance of changes in skeletal muscle mass during treatments among bladder cancer patients, further studies are needed to confirm this finding. Therapeutic interventions for cancer might improve cancer cachexia and sarcopenia by eradicating cancer cells. Because adipose tissue depletion precedes skeletal muscle wasting in animal models with cancer cachexia [26], changes in fat and skeletal muscle mass and their patterns might be associated with survival of cancer-bearing patients. Indeed, patterns of fat and skeletal muscle wasting can be associated with survival of patients with pancreatic cancer [57].

## 8. Therapeutic Interventions for Sarcopenia

Given the prognostic significance of sarcopenia and changes in skeletal muscle mass in bladder cancer patients, prevention of or recovery from sarcopenia and cancer cachexia may contribute to improving their prognosis. There are several studies to investigate nutritional support, medication, and exercise as therapeutic interventions for cancer cachexia and sarcopenia in cancer-bearing patients. However, these current therapies, such as protein supplementation, are limited due to their inefficient efficacy for improving sarcopenia, suggesting the possibility of irreversible damage to skeletal muscles [32].

### 8.1. Nutritional Support

Several previous studies show the effect of nutritional support on sarcopenia in non-cancer patients. Because sarcopenia results from a decrease in protein synthesis and increase in protein degradation, protein supplementation can play a key role in nutritional support [58]. The effects of protein supplementation on skeletal muscle mass can be increased by adding anabolic agents such as growth hormones and testosterone [58]. However, accumulating evidence suggested that sarcopenia is not fully reversed by conventional nutrition in patients with cancer cachexia [32].

### 8.2. Medications

#### 8.2.1. *n*-3 Fatty Acids

*n*-3 Fatty acids, including eicosapentaenoic acid and docosahexaenoic acid, can recover a cancer-induced hyper-catabolic state and improve sarcopenia and cachexia by its anti-inflammatory effects, involving the attenuation of NF-kB signaling, deceleration of the ubiquitin proteasome pathway, and antagonization of superoxide dismutase [59,60,61]. *n*-3 Fatty acids also reduce the expression of AZGP1 by interfering glucocorticoid receptor [32]. 4-Hydroxyhexenal (HHE) and 4-hydroxynonenal (HNE), lipid peroxidation products of *n*-3 fatty acids, can prevent the blocking of myosin expression and myotube formation caused by tumor cells [62]. *n*-3 Fatty acids can mediate the induction of apoptosis and the reduced proliferation of tumor cells [63]. Moreover, *n*-3 fatty acids have some effects on improving protein anabolism by activating the PI3K/Akt/mTOR pathway [64]. Recently, a randomized controlled study revealed that eicosapentaenoic acid improved postoperative survival in patients undergoing metastasectomy for liver metastases from colorectal cancer [65].

#### 8.2.2. ActR2B Antagonist

ActR2B, a receptor for myostatin and activin A, mediate skeletal muscle protein degradation [31]. Expression of a dominant negative ActR2B in transgenic mice leads to skeletal muscle hypertrophy, which indicates that the ActR2B pathway mediates skeletal muscle growth [66]. In mice models with cancer cachexia, blockage of the ActR2B pathway suppressed skeletal muscle wasting by abolishing activated ubiquitin-proteasome system and inducing atrophy-specific ubiquitin ligases in skeletal muscles, in which tumor growth and fat loss were not inhibited [67]. Therefore, ActR2B antagonism has therapeutic potential for treating cancer cachexia and sarcopenia.

#### 8.2.3. Medications for Insulin Resistance

Insulin resistance is basically enhanced in patients with cancer cachexia, despite the significant loss of adipose tissue [31]. Insulin resistance is related to the reduction of muscle glucose uptake and suppression of protein anabolism. Several medications for diabetes mellitus can be effective for cancer cachexia and sarcopenia. Metformin increases food intake and prolongs survival in cachectic tumor-bearing rat models [68]. Peroxisome proliferator activated receptor γ (PPAR-γ) agonists, including rosiglitazone, troglitazone, and pioglitazone, improve body weight and reduce skeletal muscle protein degradation by enhancing insulin sensitivity [69].

#### 8.2.4. Inhibitors for Lipolysis and Fatty Acid Oxidation

ATGL and HSL regulate lipolysis of triglyceride and their inhibition reduces adipose tissue loss, contributing to maintained skeletal muscle mass [29]. Therefore, inhibitors for ATGL or HSL can be candidates for medications for cancer cachexia and sarcopenia. Moreover, increased oxidative stress caused by excessive fatty acid oxidation leads to skeletal muscle wasting [30]. Therefore, etomoxir, an inhibitor of fatty acid oxidation, can be a new approach for treating cancer cachexia and sarcopenia.

#### 8.2.5. Hormonal Replacement Therapy

Hormonal replacement therapy is used to relieve menopausal symptoms in females. Because estrogen monotherapy can induce endometrial hyperplasia and cancer, progestogens are usually combined with estrogens. Hormonal replacement therapy is also effective for sarcopenia, which is one of the menopausal symptoms [70]. In males, testosterone is usually administered as hormonal replacement therapy. Testosterone improves sarcopenia, especially in combination with protein supplementations [58].

### 8.3. Exercise

Exercise, including aerobic exercise and resistance training, can contribute to the improvement of sarcopenia in cancer-bearing patients [71,72]. Exercise can overcome sarcopenia by abrogating systemic inflammation and catabolism [73]. Exercise has been reported to contribute to maintaining skeletal muscle mass and function in breast cancer patients treated with systemic chemotherapy [74]. Although the effect of exercise on metabolisms in patients with cancer cachexia is unclear, exercise may have an influence on lipolysis and insulin sensitivity. In addition, several studies demonstrated the anti-tumor effects of exercise. Exercise was shown to induce the secretion of interleukin-6 from muscles and elicit anti-tumor immunity in combination with epinephrine by redistributing natural killer cells to tumor microenvironments [75]. Because exercise enhances anti-tumor immunity, exercise might have a favorable effect on the efficacy of immune-oncology drugs for cancer [76]. Moreover, exercise was shown to inhibit tumor growth by activating the Hippo tumor suppressor pathway via β-adrenergic signaling [77].

## 9. Future Perspectives

Future studies are expected to clarify how cancer cachexia and sarcopenia progress in bladder cancer patients. Because the loss of adipose tissue precedes skeletal muscle wasting in animal models of cancer cachexia [26], phenotypes of cancer cachexia manifestations can be evaluated using CT images according to the level of fat and skeletal muscle depletion and may be useful in the management of bladder cancer patients [57]. Metabolic and molecular backgrounds of cancer cachexia and sarcopenia should be further elucidated to develop novel therapeutic strategies for sarcopenia. Future clinical trials are expected to assess the efficacy of novel medications and exercise in bladder cancer patients.

## 10. Conclusions

In this review, we summarized reported series of the prognostic role of sarcopenia in bladder cancer patients. Sarcopenia is significantly associated with unfavorable prognosis in bladder cancer patients undergoing a radical cystectomy. Moreover, sarcopenia is also a significant poor prognostic factor in patients with inoperable advanced bladder cancer. Thus, sarcopenia can be used as a prognostic biomarker in patients with bladder cancer at various stages. We reviewed reported series of the prognostic role of changes in skeletal muscle mass during treatments in bladder cancer patients. Recovery of skeletal muscle mass during treatments can be associated with the improved prognosis of bladder cancer patients, whereas decline of skeletal muscle mass can reflect poor prognosis, indicating its role not only as a prognostic biomarker but also as a surrogate marker for treatment efficacy in bladder cancer patients. In addition, nutritional support, medications, and exercise may improve sarcopenia and cancer cachexia and have a favorable influence on the management of cancer-bearing patients. Future studies may clarify the prognostic value of these interventions in cancer-bearing patients.

## Figures and Tables

**Table 1:** Reported series of the prognostic role of sarcopenia in bladder cancer cancers.

Study (Year)	Country	No. of Total Patients	No. of Sarcopenic Patients	Cancer Type	Therapeutic Interventions	Definition of Sarcopenia	Main Findings	Ref.
Smith et al. (2014)	United States	200	77 (39%)	Bladder cancer	Radical cystectomy	TPA ^†^ < 653 cm^2^/m^2^ for males and <523 cm^2^/m^2^ for females	The Kaplan–Meier curves showed no significant association between OS and sarcopenia (*p* = 0.36).	[45]
Psutka et al. (2014)	United States	205	141 (69%)	Bladder cancer	Radical cystectomy	SMI < 55 cm^2^/m^2^ for males and <39 cm^2^/m^2^ for females	Sarcopenia was an independent poor prognostic factor with HR 2.14 for CSS (*p* = 0.007) and 1.93 for OS (*p* = 0.004).	[46]
Hirasawa et al. (2016)	Japan	136	65 (48%)	Bladder cancer	Radical cystectomy	SMI < 43 cm^2^/m^2^ for males with BMI < 25 cm^2^/m^2^, <53 cm^2^/m^2^ for males with BMI ≥ 25 cm^2^/m^2^, and <41 cm^2^/m^2^ for females	Sarcopenia was an independent poor prognostic factor with HR 2.3 for CSS (*p* = 0.015).	[47]
Miyake et al. (2017)	Japan	89	22 (25%)	Bladder cancer	Radical cystectomy	SMI < 43 cm^2^/m^2^ for males with BMI < 25 cm^2^/m^2^, <53 cm^2^/m^2^ for males with BMI ≥ 25 cm^2^/m^2^, and <41 cm^2^/m^2^ for females	Sarcopenia was an independent poor prognostic factor with HR 2.2 for OS (*p* = 0.03).	[48]
Saitoh-Maeda et al. (2018)	Japan	63 (male only)	141 (69%)	Bladder cancer	Radical cystectomy	PMI < 400 cm^2^/m^2^	In male patients, non-sarcopenic patients had a significantly better OS than sarcopenic counterparts (2,889 vs. 2,009 days; *p* = 0.023).	[49]
Mayr et al. (2018)	Germany	500	189 (38%)	Bladder cancer	Radical cystectomy	SMI < 43 cm^2^/m^2^ for males with BMI < 25 cm^2^/m^2^, <53 cm^2^/m^2^ for males with BMI ≥ 25 cm^2^/m^2^, and <41 cm^2^/m^2^ for females	Sarcopenia was an independent poor prognostic factor with HR 1.42 for CSS (*p* = 0.048) and 1.43 for OS (*p* = 0.01).	[50]
Fukushima et al. (2015)	Japan	88	67 (76%)	Advanced urothelial carcinoma	Miscellaneous	SMI < 43 cm^2^/m^2^ for males with BMI < 25 cm^2^/m^2^, <53 cm^2^/m^2^ for males with BMI ≥ 25 cm^2^/m^2^, and <41 cm^2^/m^2^ for females	Sarcopenia was an independent poor prognostic factor with HR 3.36 for OS (*p* < 0.001).	[51]
Taguchi et al. (2015)	Japan	100	Not reported	Metastatic urothelial carcinoma	Systemic chemotherapy	SMI < 55 cm^2^/m^2^ for males and <39 cm^2^/m^2^ for females	Sarcopenia was an independent poor prognostic factor with HR 2.07 for CSS (*p* = 0.045).	[52]
Kasahara et al. (2017)	Japan	27	14 (52%)	Advanced bladder cancer	Systemic chemotherapy	PMI < 2.49 cm^2^/m^2^ for males and <2.07 cm^2^/m^2^ for females	The OS of the non-sarcopenic group was significantly better than that of the sarcopenic group (561 vs. 223 days; *p* = 0.0150).	[53]
Abe et al. (2018)	Japan	87	Not reported	Metastatic urothelial carcinoma	Systemic chemotherapy	SMI < 55 cm^2^/m^2^ for males and <39 cm^2^/m^2^ for females	Sarcopenia was not significantly associated with OS (*p* = 0.11). SMI stratified by BMI was an independent predictor for shorter OS (*p* = 0.026).	[54]

Abbreviations: BMI = body mass index; CSS = cancer-specific survival; HR = hazard ratio; OS = overall survival; PMI = psoas muscle index; SMI = skeletal muscle index; TPA = total psoas area. ^†^ TPA was calculated by measuring the cross-sectional area of the right and left psoas muscles on CT using 3-dimensional computerized image analysis.

**Table 2:** Reported series of the prognostic role of changes in skeletal muscle mass in bladder cancer cancers.

Study (Year)	Country	No. of Total Patients	Cancer Type	Therapeutic Interventions	Evaluation of Skeletal Muscle Mass	Main Findings	Ref.
Miyake et al. (2017)	Japan	89	Bladder cancer	Radical cystectomy	Postoperative changes in psoas major muscle volume were calculated after a radical cystectomy.	A 10% loss in the volume of the psoas muscle was an independent poor prognostic factor with HR 2.4 for OS (*p* = 0.02).	[48]
Zargar et al. (2017)	United States	60	Bladder cancer	NAC and a radical cystectomy	Changes in PMV were calculated from pre- and post-NAC CT images.	The proportion of PMV decline during NAC was not a predictor of OS after a radical cystectomy (*p* = 0.85).	[55]
Fukushima et al. (2018)	Japan	72	Advanced urothelial carcinoma	Systemic chemotherapy	Changes in SMI were calculated from pretherapeutic and posttherapeutic CT images.	Post-therapeutic skeletal muscle mass recovery was an independent favorable prognostic factor with HR 0.24 for RFS (*p* < 0.001) and 0.21 for OS (*p* < 0.001).	[56]

Abbreviations: CT = computed tomography; HR = hazard ratio; NAC = neoadjuvant chemotherapy; OS = overall survival; PMV = psoas muscle volume; RFS = recurrence-free survival; SMI = skeletal muscle index.
